# An Alternative Vaccination Approach for The Prevention of Highly Pathogenic Avian Influenza Subtype H5N1 in The Red River Delta, Vietnam—A Geospatial-Based Cost-Effectiveness Analysis

**DOI:** 10.3390/vetsci3010006

**Published:** 2016-02-06

**Authors:** Chinh C. Tran, John F. Yanagida, Sumeet Saksena, Jefferson Fox

**Affiliations:** 1 Department of Natural Resources and Environmental Management, University of Hawaii at Manoa, 1910 East-West Road, Honolulu, HI 96822, USA; jyanagid@hawaii.edu; 2 East-West Center, 1601 East-West Road, Honolulu, HI 96848, USA; saksenas@eastwestcenter.org (S.S.); foxj@eastwestcenter.org (J.F.)

**Keywords:** HPAI H5N1, alternative vaccination program, national poultry vaccination program, Red River Delta

## Abstract

This study addresses the tradeoff between Vietnam’s national poultry vaccination program, which implemented an annual two-round HPAI H5N1 vaccination program for the entire geographical area of the Red River Delta during the period from 2005–2010, and an alternative vaccination program which would involve vaccination for every production cycle at the recommended poultry age in high risk areas within the Delta. The *ex ante* analysis framework was applied to identify the location of areas with high probability of HPAI H5N1 occurrence for the alternative vaccination program by using boosted regression trees (BRT) models, followed by weighted overlay operations. Cost-effectiveness of the vaccination programs was then estimated to measure the tradeoff between the past national poultry vaccination program and the alternative vaccination program. *Ex ante* analysis showed that the focus areas for the alternative vaccination program included 1137 communes, corresponding to 50.6% of total communes in the Delta, and located primarily in the coastal areas to the east and south of Hanoi. The cost-effectiveness analysis suggested that the alternative vaccination program would have been more successful in reducing the rate of disease occurrence and the total cost of vaccinations, as compared to the national poultry vaccination program.

## 1. Introduction

The Highly Pathogenic Avian Influenza (HPAI) subtype H5N1 has had serious, detrimental effects on the economy and human health in Vietnam since the first reported outbreak on 27 December 2003 [1]. Millions of poultry were culled due to disease occurrences, causing an estimated economic loss of 3 trillion VND (approximately U.S. $187.15 million) [2,3]. The average growth rate of poultry population was reduced from 7.6% for the period 2000–2003 (before HPAI H5N1 occurrence) to 3.8% for the period 2003–2006 (during HPAI H5N1 disease occurrence) [4]. Market demand and price decreases caused further economic losses to poultry producers [5,6]. The disease also seriously affected human health. By 19 November 2010, a total of 119 human cases of HPAI H5N1 were reported, with 59 deaths [7].

Financial support from many international organizations, including the Food and Agriculture Organization (FAO) of the United Nations, the World Bank, the World Health Organization, and others, helped to contain the disease as designed in the Integrated National Operational Program for Avian and Human Influenza (OPI) which is also known as the *Green Book* [8]. The overall objective of the OPI was to reduce the health risk to humans by controlling the HPAI H5N1 disease at the source in domestic poultry. The national poultry vaccination campaign was a key strategic plan in the OPI [8] (we hereby use the term “national poultry vaccination program” throughout the study). After the pilot vaccinations implemented in Nam Dinh and Tien Giang provinces in August 2005, a mass vaccination campaign was conducted nationally from late September to the beginning of November, 2005 and continued until the end of 2010. The vaccination was applied nationally in two rounds per year with the first round from April–May and the second round from October–November. The vaccination program continued in later years, but on a smaller scale, and was primarily determined and implemented by provincial authorities. 

Vaccination has been shown to be a viable means of protection against the HPAI H5N1 virus [9]. While the campaign was carried out only twice a year in April–May and October–November, poultry production, however, occurs all year round. A previous study confirmed that November to January and April to June were the periods that are most vulnerable for disease occurrence [5]. A sizeable proportion of poultry population remained unvaccinated at different times of the year. Therefore, unvaccinated poultry between the two rounds of vaccinations will be at risk of infection. As a result, the disease has been repeatedly reported over the years. Circulation of the HPAI H5N1 virus was found mostly in unvaccinated waterfowl, especially ducks [10,11]. Additionally, the cost of the vaccination program was estimated to be approximately U.S. $10 million per round [7,12,13].

Therefore, it is critical to explore implications of an alternative vaccination program, which is likely to be more successful in containing and preventing the disease from recurrence in the Red River Delta, Vietnam, and reducing total vaccination costs as compared to the national poultry vaccination program implemented in the period 2005–2010. This study focuses on the Red River Delta of Vietnam. This area has been identified as a high-risk area for the disease [10,14]. Vaccination was implemented across all geographical areas for all provinces in the Delta under the national poultry vaccination program. 

A number of studies have identified factors affecting the occurrence and spread of the HPAI H5N1 virus in Vietnam, in general, and in the Red River Delta, in particular. It was suggested that higher average monthly temperatures and poultry density in combination with lower average monthly precipitation, humidity, and elevation significantly affected the occurrence of HPAI H5N1 in the Delta [5]. Other factors linked with the disease at the national level were a higher proportion of land used for rice paddy fields and aquaculture, increases in production, trade and movement of live poultry, and the expansion of free-grazing ducks [14,15,16,17]. 

Given these findings, it is not likely that all areas within the Red River Delta are equally susceptible to the disease. We propose an alternative vaccination program which involves shifting from a less frequent blanket vaccination for the entire Delta to more frequent vaccination in high probability areas for disease occurrence within the Delta. This modification would involve vaccination for every production cycle at the recommended poultry age in high risk areas. Two key questions emerge with this proposal: (i) Where are the higher probability areas (focus areas) for the alternative vaccination program? (ii) Is it beneficial for the Government to switch to the alternative vaccination program in terms of the cost-effectiveness of vaccination programs? To answer questions and fulfill the objective, this study (i) identifies the focus areas for the alternative vaccination program to be implemented in the Red River Delta, and (ii) estimates the tradeoff between the national poultry vaccination program and the alternative vaccination program based on the cost-effectiveness analysis of vaccination programs.

## 2. Methods

### 2.1. Study Area and Data Sources 

**Figure 1:**
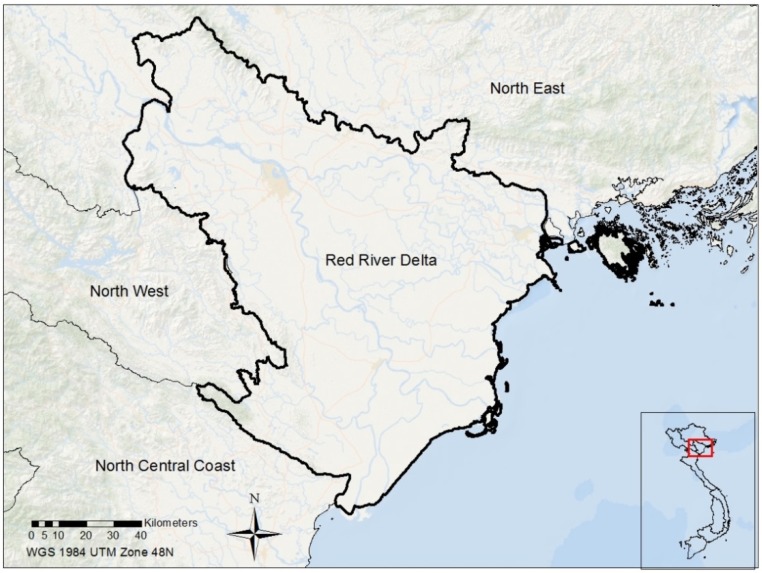
The study area—the Red River Delta, Vietnam.

This study focuses on the Red River Delta of Vietnam (Figure 1), which represents one of the two largest flood plains in Vietnam. The Delta includes two large river systems—the Red river and Thai Binh river systems—that support agricultural and livestock activities. The Red River Delta includes eight provinces and two municipalities, the capital city of Hanoi and the main port of Hai Phong. The Delta plays an important role and interacts with a wide range of environmental and socioeconomic sectors, including industry, commerce, services, agriculture, tourism, *etc.* Livestock production is among the main activities in the Delta, including poultry, pig, and cow husbandry. Poultry production has faced serious problems caused by the HPAI H5N1 disease.

The HPAI H5N1 outbreak data are routinely collected and reported by the Vietnam Department of Animal Health. The Red River Delta has been identified as a high-risk area for the disease [10,14]. The Delta has been severely affected by three out of the five large epidemic waves of HPAI H5N1 outbreaks reported in Vietnam. These three waves included the first wave, which occurred from December 2003–February 2004, the third wave was recorded from October–December 2005, and the fifth wave reported from May–September 2007 [14,18]. A number of outbreaks in the second epidemic wave which was from November 2004 to March 2005 were also reported in the Red River Delta despite the primary effects occurring in the Mekong Delta. Other sporadic outbreaks occurred over the period from December 2003 to the present, with recent reported outbreaks in Bac Ninh and Nam Dinh provinces in January and February 2014 [1]. Although the disease occurred in the Delta with the first epidemic wave reported from the end of 2003, the dates and locations of occurrences were not formally reported until the end of March 2004 [14]. Therefore, this study analyzed reported disease data for the period starting from the end of March, 2004, to the end of December, 2007, which included four large epidemic waves of HPAI H5N1 outbreaks with 267 confirmed HPAI H5N1 outbreaks in the Red River Delta. The data were reported at the commune level and coded as 1 if the disease was found or 0 if there was no disease reported.

Other risk factors used for the analysis were identified based on earlier studies of HPAI H5N1 in Vietnam. These included the percentage of land used for rice paddy fields and aquaculture [14,19], chicken and water bird density [4,14,15] and elevation [5,14,17]. Land use diversity was found to be significantly associated with disease occurrence [19]. Land use/land cover is dominated by forests and permanent vegetation in high elevation areas, and characterized by agriculture and a mixed uses of land in low elevation areas [17]. Disease occurrence was also found around heavily-populated cities in different regions in Vietnam such as Ho Chi Minh city in the South, Da Nang city in the central part, and Hanoi and Hai Phong cities in the north [14]. Therefore, two other land use factors, characterizing built-up and forest/perennial trees features, were also included. 

**Figure 2:**
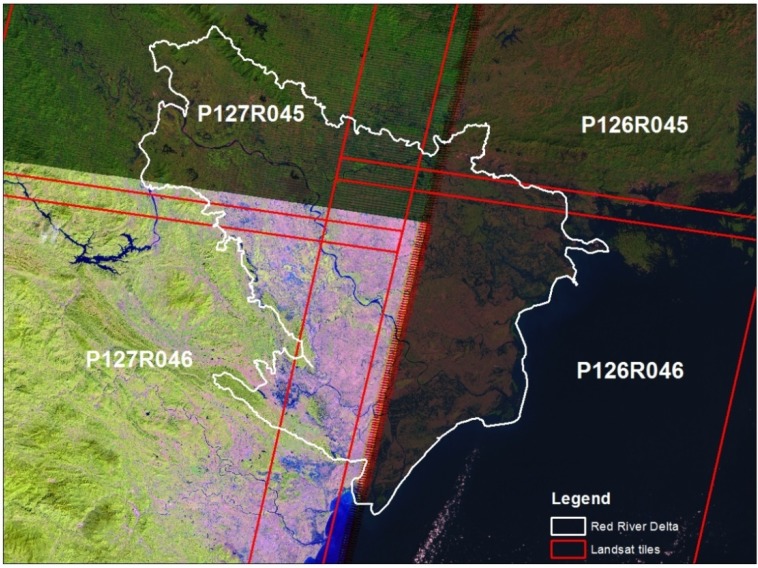
Landsat tiles covering the Red River Delta of Vietnam.

These variables, percentage of land use for rice paddy fields, aquaculture, built-up and forest/perennial trees, and chicken and water bird density, were measured at the commune level and obtained from The 2006 Vietnam Rural, Agricultural and Fishery Census provided by the East West Center–National Science Foundation project, “Coupled Natural-Human Systems and Emerging Infectious Diseases: Anthropogenic environmental change and avian influenza in Vietnam”. Elevation data were obtained from the Shuttle Radar Topography Mission (SRTM) 90-m resolution Digital Elevation Model (DEM) [20]. These data were then retrieved for each commune and merged with other data using commune codes for the statistical analysis. Remotely sensed Landsat TM/ETM+ Bands 1–5, 7 data that cover the study area were downloaded from the USGS EROS Data Center [21]. The Red River Delta is covered by four Landsat tiles: P126R045, P126R046, P127R045, and P127R046 (Figure 2). 

### 2.2. Statistical Analysis

For the first objective involving identification of focus areas for the alternative vaccination program in the Red River Delta, this study adopted the *ex ante* analysis framework. Outbreak data were divided into two datasets. The first dataset contained the data before the launch of the national poultry vaccination program in 2005, which included the second and the third epidemic waves, with a total of 193 outbreaks. The second dataset consisted of outbreak data that occurred after 2005 which comprised the fifth epidemic wave with 74 outbreaks. The use of the first dataset was to identify focus locations for the alternative vaccination program. The second dataset would be used to evaluate the cost and effectiveness of the vaccination program in analyzing the tradeoff between the national poultry vaccination program and alternative vaccination program.

#### 2.2.1. *Ex Ante* Analysis

For *ex ante* analysis, weighted overlay analysis was applied to identify the focus locations for the alternative vaccination program. This method has been considered as one of the most suitable techniques and is frequently used for site selection and suitability models in spatial analysis [22]. It has been widely applied in several fields, e.g., disease management, climate change, habitat conservation, sustainable ecosystems, and land-use planning, *etc.* [23,24,25,26,27]. This technique requires that all input factors are classified into different groups and weighted to determine their weight accordingly. The analytical procedure for weighted overlay analysis in this study involved a two-stage process: (1) boosted regression trees (BRT), followed by (2) weighted overlay operations. 

The first step is to run BRT to determine the appropriate weighting for the main factors that were previously found to have significant effects on disease occurrence, including water bird density, chicken density, elevation, and land use/land cover (Table 1). BRT utilizes a combination of decision trees and boosting algorithms to improve prediction accuracy through an iterative process [28,29]. This method has been popularly applied for predicting the distribution of HPAI H5N1 disease [19,30,31,32,33]. Several combinations of the learning rate and tree complexity were tested to choose the best setting for evaluating model performance which was determined through cross-validation (CV). The final model setting was the combination of a tree complexity of 4 and a learning rate of 0.005, with a bag fraction of 0.75, which were also previously used in [30]. The results present which factors appear to have more influence on the disease occurrence in the Red River Delta.

**Table 1:** Critical factors affecting the disease occurrence.

Factors	Data Sources	Attribute Values of Factors
Water bird density (heads/km^2^)	The 2006 Vietnam Rural, Agricultural and Fishery Census	0–892
		893–2097
		2098–4299
		>4299
Chicken density (heads/km^2^)	The 2006 Vietnam Rural, Agricultural and Fishery Census	0–1738
		1739–3992
		3993–9472
		>9472
Elevation (m)	SRTM 90-m resolution DEM: http://srtm.csi.cgiar.org/ [20]	≤5
		>5–15
		>15–200
		>200
Land use/land cover (%)	The 2006 Vietnam Rural, Agricultural and Fishery Census	Agriculture
		Aquaculture
		Built-up
		Forest/perennial trees

Each factor has its own characteristics that also have impacts on the disease. The next step is to measure the weights of attribute values of each factor. The land use/land cover included land for agriculture, aquaculture, built-up purposes, and forest/perennial tree areas. The Red River Delta topography was reclassified into four groups of elevation (above 200 m, from 15 m to 200 m, from 5 m to 15 m, and less than 5 m) and coded from 1 to 4, respectively, to represent upland, midland, lowland, and coastal areas (see [5]). Other factors were categorized into four groups by using the commonly-used Jenks natural breaks classification method [34] in ArcGIS to group data into categories (Table 1). 

The second step determined the potential focus area for the vaccination program by performing weighted overlay analysis in ArcGIS 10.1 (ESRI, Redlands, CA, USA). The analysis was operated in raster layers. Therefore, vector layers detailing water bird density and chicken density at the commune level were converted to corresponding raster layers. The elevation data were originally stored in raster format. For the spatial distribution of land use/land cover, a support vector machine (SVM) was applied to classify remotely-sensed imagery data in the Red River Delta into four categories that represent built-up, agriculture, forest/perennial trees, and water areas. The SVM method has been successfully applied in several studies on biophysical tasks, land cover/land use including vegetation, agriculture, and impervious surfaces, such as urban areas, *etc.* [35,36,37]. The classification process was performed using ENVI version 4.8 (Exelis Visual Information Solutions, Boulder, CO, USA) and ArcGIS version 10.1 (ESRI, Redlands, CA, USA). Outputs were the classification maps of land use/land cover for each subset and were mosaiced together to produce the final land use/land cover classification map for the Red River Delta.

All raster layers were then reclassified using the corresponding weight obtained from BRT results for the weighted overlay operations processed in ArcGIS version 10.1 to find the suitable areas for the alternative vaccination program. The analysis provides suitability maps with suitability scores in integer numbers scaled from 0 to 100. The higher suitability scores represent the higher probability of contracting the HPAI H5N1 disease. Areas with higher suitability scores are suggested as good candidates where the alternative vaccination program should be focused.

#### 2.2.2. The Cost-Effectiveness Analysis

For the tradeoff between the past national poultry vaccination program and the alternative vaccination program, the cost and effectiveness of the vaccination program were estimated for each program. The second dataset which included data for the fifth epidemic wave with 74 affected communes was used in addition to the first dataset. To implement the vaccination program, the government is responsible for all the costs, including the costs of vaccine, labor, and other costs associated with vaccination. The costs for the vaccination program are the product of the number of birds vaccinated, the cost of vaccination per bird, and the number of vaccination rounds per year. When the disease occurs, the Vietnamese government implements the stamping out method, which culls all birds in affected communes and emergency vaccination is deployed to vaccinate all birds in surrounding communes in order to contain and prevent the disease from spreading. These are extra costs of the vaccination program. The total cost of the vaccination program is comprised of the cost of the government vaccination program, the cost of emergency vaccination, government compensation for birds culled, and farmer's losses because of the value difference between market price and the government's compensation when the disease occurs.

(1) Cost=A×C×N+B×C+I×G+(P−G)×I

where A is the number of birds vaccinated; C is the costs of vaccination per bird; N is the number of vaccination rounds per year; B is the number of birds vaccinated because of emergency vaccination; I is the number of birds culled due to the disease occurrence; G is the government compensation per bird culled; and P is the market price per bird.

The effectiveness of the vaccination program is the measure of proportionate reduction in the rate of disease occurrence as the result of the vaccination program. This can be achieved through the calculation based on the relative risk of the disease [38].

(2) $$\text{Effectiveness}=\frac{\text{ARU-ARV}}{\text{ARU}}\times 100$$

where ARU and ARV are, respectively, the infection rates before and after the launch of the vaccination program. The infection rate is the number of affected communes divided by the total communes in the Red River Delta.

## 3. Results and Discussion

### 3.1. Results

#### 3.1.1. *Ex Ante* Analysis

The BRT results (see Table 2) suggest that water bird density had the largest effect on disease occurrence with the weight estimated at 19%. Ducks, as a reservoir host for the HPAI H5N1 virus, have been discussed in earlier studies [10,16,17,39,40,41,42]. The number of recorded duck-related disease occurrences steadily increased from 11% in 2003/2004 to its peak of 78% in 2006/2007 [10]. The next highest weight factors were land used for rice paddy field, elevation, land used for aquaculture, land used for built-up purposes, chicken density, and land used for forest/perennial trees. Land used for forest/perennial trees had the smallest effect with 2% weight. 

**Table 2:** Estimated weight of each factor to the HPAI H5N1 occurrences.

Variable	Weight (%)
Water bird density	19
Land used for rice paddy field	18
Elevation	18
Land used for aquaculture	17
Land used for built-up purposes	14
Chicken density	12
Land used for forest/perennial trees	2

Table 3 reveals the weight of attribute values of each factor; lower water bird density and chicken density were found to have higher weight as compared to other groups within each factor. Specifically, water bird density ranging from 893–2097 heads/km^2^ had the highest weight of 76% for the water bird density factor. Chicken density attributes ranging from 0–1738 heads/km^2^ showed the highest weight of 37%. Elevation less than 5 m was estimated to have a 73% weight. These were flat plain areas where rice production was the predominant agricultural activity in the Red River Delta. Lowlands with elevation ranging from 5–15 m, and midland areas with elevation ranging from 15–200 m, were ranked the second and third with 26% and 11% weights, respectively. These areas were located to the west of the Delta, including the capital city of Hanoi. Land used for rice production also had the highest contribution to disease occurrence with a 46% weight and followed by land used for built-up purposes at 35%.

**Table 3:** Attribute weight of each group within a factor.

Factors	Attribute Values of Factors	Attribute Weight (%)
Water bird density (heads/km^2^)	0–892	11
	893–2097	76
	2098–4299	7
	>4299	6
Chicken density (heads/km^2^)	0–1738	37
	1739–3992	27
	3993–9472	25
	>9472	11
Elevation (m)	≤5	73
	>5–15	16
	>15–200	11
	>200	0
Land use/land cover (%)	Agriculture	46
	Built-up	35
	Aquaculture	17
	Forest/perennial trees	2

**Figure 3:**
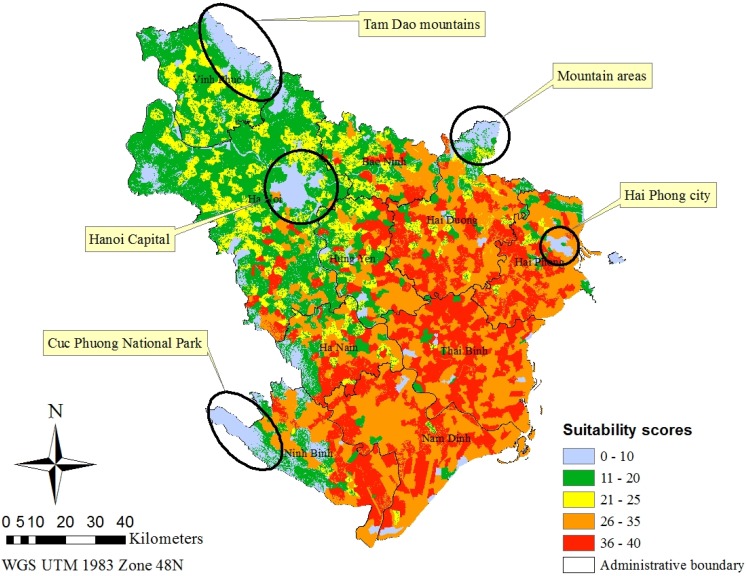
Weighted overlay analysis results for the HPAI H5N1 occurrence in the Red River Delta.

The BRT estimation results provided essential information for weighted overlay analysis. The weight of each attribute in Table 3 was assigned to corresponding groups in each raster layer through raster reclassification processes. For instance, in the land-use raster, agriculture, built-up, water, and forest/perennial trees groups were, respectively, assigned their corresponding weight values of 46, 35, 17, and 2 (Table 3). The same procedure was applied to other raster layers detailing water bird density, chicken density, and elevation. Each of the input rasters was then weighed using the weighted values from Table 2. In this weighted overlay analysis, water bird density had a 19% weight, land used for rice paddy field an 18% weight, elevation an 18% weight, land used for aquaculture a 17% weight, land used for built-up purposes a 14% weight, chicken density a 12% weight, and land used for forest/perennial trees a 2% weight. The output suitability map is shown in Figure 3.

The highest suitability score areas were shown in red, followed by orange. Yellow, green, and blue areas have lower suitability scores. These areas (suitability scores ranging from 0–10, 11–20, and 21–25, respectively) were mostly located to the west and northwest of the Red River Delta. The areas with high suitability scores (ranging from 26–40), as shown in red and orange, were chosen as the focus areas for the alternative vaccination program against HPAI H5N1. 

#### 3.1.2. Cost-Effectiveness Analysis of Vaccination Programs and Policy Implications 

**Figure 4:**
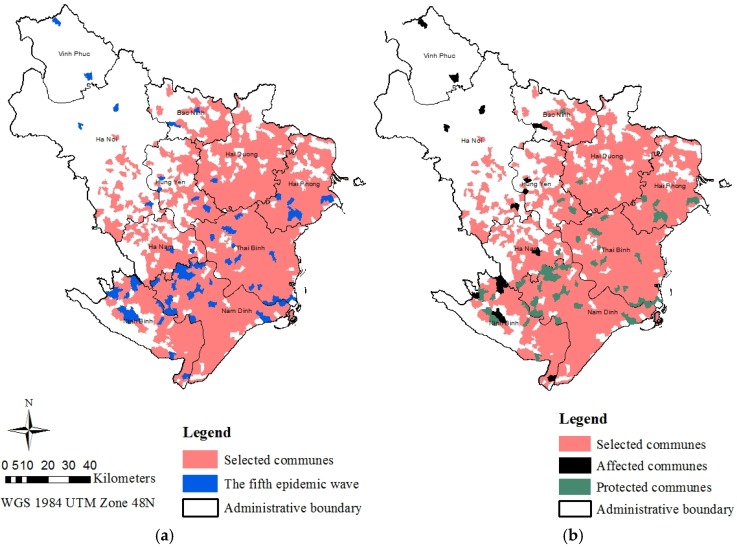
(**a**) Spatial distribution of the fifth epidemic wave in the Red River Delta and the focus areas for the alternative vaccination program; and (**b**) spatial distribution of the estimated affected communes and protected communes under the alternative vaccination program.

The focus areas for the alternative vaccination program were extracted and overlaid with the spatial distribution of the fifth HPAI H5N1 (Figure 4a) for the cost-effectiveness analysis. The fifth wave of outbreak, with 74 affected communes, was the result of the national poultry vaccination program. Based on the Vietnam follow-up disease report No. 45 released by OIE (The World Organisation for Animal Health) for the period from December 2006 to December 2010 [43], we found that the disease was mostly reported in unvaccinated poultry. Vaccines mainly used in Vietnam contain a killed antigen combined with an oil-based adjuvant: (1) A/TK/England/N-28/73, subtype H5N2 (referred to as N28); and (2) a genetically modified reassortant H5N1 low-pathogenic virus, A/Harbin/Re-1/2003 (referred to as Re-1) [44]. Vaccines were found to provide protection against the disease and reduction of viral shedding on both chickens and ducks [44]. The vaccination program proved to be a viable means of protection against the HPAI H5N1 virus [9]. Given this information, this study assumed 100% vaccine efficacy in order to evaluate the tradeoff in cost-effectiveness between the two vaccination programs. As a result, it is expected that the focus areas are protected from the HPAI H5N1 disease as the result of frequent vaccinations under the alternative vaccination program. Figure 4b showed that a total of 61 out of 74 infected communes in the fifth epidemic was correctly predicted in the focus areas for the alternative vaccination program. As a result, the alternative vaccination program would protect these 61 communes from the disease. However, the other 13 communes which were not covered by this program could be affected by the disease.

The cost-effectiveness analysis of the vaccination programs was conducted by using Equations (1) and (2) to investigate which program would be more successful in preventing the disease occurrence. The results of the analysis are shown in Table 4. 

Of the total number of 2248 communes in the River Delta, there were 193 communes affected by the disease before the implementation of the national poultry vaccination program. This resulted in the infection rate ARV = 8.59. The national poultry vaccination program contributed to the reduction of the affected communes to 74 as reported in the fifth epidemic waves, resulting in the infection rate ARU = 3.29. The alternative vaccination program was expected to further reduce the number of communes affected to 13 communes which yields the infection rate ARU = 0.58. The effectiveness results suggested that the alternative vaccination program would be more successfully in reducing the rate of disease occurrence measured at 93.26%, compared to the national poultry vaccination program at 61.66%. 

It was previously estimated that the optimal length of both chicken and duck production cycles were 10 weeks, including a two-week cleaning period [45,46]. Assuming that producers are continuously engaged in production, there would be five duck production cycles per year. Therefore, the alternative vaccination program would involve five vaccination campaigns throughout the year. It was noted that the national poultry vaccination program covered the entire poultry population in the Red River Delta and was conducted twice a year. The cost of a HPAI H5N1 vaccination in Viet Nam was estimated to be U.S. $0.038/head, including the vaccine cost of U.S. $0.016 per dose, labor cost of U.S. $0.013, and other costs associated with vaccination of U.S. $0.009 [47]. As a result, the cost of the national poultry vaccination program was estimated at U.S. $4.50 million per year. The alternative vaccination program consists of about half of the total communes in the Delta, covering 1137 communes with poultry population of 31,171 thousand birds. Having vaccinated poultry five times per year would cost U.S. $5.92 million per year. By examining the costs of vaccination only, the cost of the alternative vaccination program appears to be higher than the national poultry vaccination program (U.S. $5.92 million *vs*. U.S. $4.50 million). 

**Table 4:** Cost-effectiveness analysis of the two programs.

Content	Before Vaccination Program	National Poultry Vaccination Program	Alternative Vaccination Program
Total number of communes involved in vaccination program	0	2248	1137
Number of rounds of vaccination per year	0	2	5
Number of birds vaccinated per round (Thousand heads)	0	59,241	31,171
Number of affected communes	193	74	13
Infection rate (%)	8.59	3.29	0.58
Effectiveness (%)		61.66	93.26
Vaccination cost (Million U.S. $)	0	4.50	5.92
Number of birds culled (Thousand heads)	6375	2165	395
Cost of culling birds (Million U.S. $)	1.59	0.54	0.10
Government compensation (Million U.S. $)	7.95	2.68	0.49
Number of communes included in emergency vaccination	543	304	92
Number of birds vaccinated in emergency vaccination (Thousand heads)	0	8625	2898
Cost of emergency vaccination (Million U.S. $)	0	0.33	0.11
Farmers loss (Million U.S. $)	4.95	1.65	0.30
Total loss (Million U.S. $)	14.49	9.70	6.92

When the disease occurs, all birds in affected communes are culled due to the stamping out program and all birds in surrounding communes are vaccinated. Before the official vaccination campaign was launched at the end of 2005, the only emergency response to the disease occurrence was the stamping out program. This resulted in 6.375 million. birds in 193 affected communes culled in the second and third epidemic waves. After the implementation of the national poultry vaccination program, a total of 2.165 million birds were affected and culled, and 8.625 million birds were vaccinated, as the result of the emergency response to the disease occurrence. Under the alternative vaccination program, it was estimated that 395,000 birds in 13 communes were affected and culled by the disease. Another 2.898 million birds in 92 surrounding communes were vaccinated due to the emergency vaccination.

The government incurred more losses from the stamping out and emergency vaccination. The cost of culling birds in the stamping out process was estimated at U.S. $0.25/head [47]. Total culling cost was estimated at U.S. $1.59 million before the implementation of the national poultry vaccination program. This cost was much smaller as a result of either the national poultry vaccination program (U.S. $0.54 million) or the proposed alternative vaccination program (U.S. $0.10 million). The average amount of compensation per bird culled due to the disease occurrence was regulated at U.S. $1.24/head (23,000 VND/head) (exchange rate at 1 USD = 18,500 VND), in Decision No 719/QD-TTg, dated 5 June 2008. In addition, the average market value of a bird was estimated at U.S. $2 [12]. The farmers also suffered losses of U.S. $0.76/head from production because of the value difference between the market price and the government compensation. This resulted in the Government's additional losses from compensation and farmers' losses of U.S. $7.95 million and U.S. $4.95 million, respectively, in the second and third epidemic waves. Under the national poultry vaccination program, emergency vaccination was implemented which caused the government an estimated U.S. $0.33 million in losses in addition to the government compensation and farmers losses measured at, respectively, U.S. $2.68 million and U.S. $1.65 million. These losses were also estimated at U.S. $0.49 million, U.S. $0.11 million, and U.S. $0.30 million for the government and farmers under the alternative vaccination program. Except for the costs of vaccination (U.S. $4.50 million *vs.* U.S. $5.92 million), and other costs, the alternative vaccination program costs were lower than the national poultry vaccination program.

Total losses imposed on the government and farmers were highest without vaccination and estimated at U.S. $14.49 million, as compared to U.S. $9.70 million for the national poultry vaccination program, and U.S. $6.92 million for the alternative vaccination program. 

### 3.2. Discussion 

A vaccination program was identified as a key strategic plan in preventing the HPAI H5N1 disease in Vietnam in the period 2006–2010 [8]. Although the implementation of the national poultry vaccination program resulted in the reduction in disease outbreaks and number of infected birds [44], the disease was repeatedly reported during this period. The third epidemic wave of outbreaks occurred during the first vaccination campaign (September–December 2005). The fourth and fifth waves occurred in between two rounds of vaccination which were from December 2006–March 2007 and May–September 2007, respectively. Infection was reported in unvaccinated ducks based on routine surveillance [10,44]. During the time between the two rounds of vaccinations, the unvaccinated poultry are susceptible to the disease. Additionally, the best vaccination immunity occurs when poultry is vaccinated at the recommended ages suggested by vaccine manufacturers [12]. The national poultry vaccination program scheduled vaccinations at a certain time in each province. It is highly unlikely that the vaccination program exactly matches the optimal timing for vaccination recommended for poultry at various ages. 

The alternative vaccination program seeks to fill this gap between the two vaccination rounds and focus more on high risk areas of disease occurrences. The alternative vaccination program was proposed to vaccinate poultry for every production cycle at the recommended poultry ages in high risk areas. Vaccinating poultry at early age provides high level of immunity [44]. A two-dose vaccine given to ducks at one day of age and a booster at four weeks of age produced effective protection [44,48]. It is critical to understand what factors potentially affect the disease occurrence in order to locate susceptible areas where the disease is likely to occur. These factors have been analyzed in previous studies. This study incorporated these factors to spatially identify the focus areas for the proposed alternative vaccination program. 

Traditional production methods with free range water bird farming and backyard chicken farming have been considered to be typical Asian production methods which had the potential of contracting and spreading the HPAI H5N1 virus to other neighboring farms [49,50]. These methods were found popular in the poultry sectors 3 and 4 (as classified by [51]) which have small scale production with less than 2000 birds [4]. Poultry sectors 1 and 2, on the other end, are characterized by industrial and commercial poultry production which operate with standard procedures and keep poultry indoors continuously during production to maintain high biosecurity standards against diseasesh, including HPAI H5N1 [51,52]. The BRT results showed the same trend. Water bird density ranging from 893–2097 heads/km^2^ and chicken density ranging from 0–1738 heads/km^2^ were found to have higher impacts on the disease occurrence as their weights were estimated at 76% for water bird density factor and 37% in water bird and chicken density factors, respectively. Therefore, it was expected that poultry sectors 3 and 4 would fall more in the lower density group. This result also agreed with the study by [9], which suggested communes with medium water bird density to have an increased risk of contracting the disease. Communes with higher water bird and chicken density would be expected to fall in poultry sectors 1 and 2, which have more secure closed farming methods against the disease than the free range farming.

Lower elevation was previously identified to be correlated with the HPAI H5N1 disease in Vietnam, in general, and in the Red River Delta, Vietnam, in particular [5,17]. This finding was further confirmed by BRT estimation. It was suggested that topographic elevation features noticeably contributed differently to the disease occurrence. Coastal areas with elevation less than 5 m contributed to the likelihood of disease occurrence. In contrast to lower elevation areas, upland areas with elevation greater than 200 m were found not likely to affect the disease. Evergreen forests or forestry production dominates these areas [53]. This was also consistent with BRT estimation for land use/land cover which showed that the weight of land used for forest/perennial trees was small and measured at 2% (see Table 2).

The BRT results for land use/land cover were in agreement with studies by [14] and [17] which suggested the link between HPAI H5N1 occurrence and the higher proportion of land use for rice paddy fields and closer distance to higher-density human population areas. Water bird movement through rice paddy fields has been defined as a potential source for spreading the HPAI H5N1 virus [10,14,16,17,54]. The built-up areas characterizes urban and peri-urban areas. It was found that peri-urban areas were the hotspot for the occurrence of the disease. These are the places where land-use changes, interaction, and contact between human and poultry become more frequent and, therefore, they were found to be at significantly higher risk for HPAI H5N1 occurrence [19]. 

The suitable areas for the vaccination campaign are shown in Figure 3. These suggested areas are characterized by low and high suitability scores. The lowest suitability score areas were either in urban cores or mountainous areas. They included urban core areas of Hanoi, Hai Phong, Hai Duong, Bac Ninh, Hung Yen, Nam Dinh, and Thai Binh provinces and mountain areas of Ba Vi of Hanoi, Tam Dao of Vinh Phuc, Cuc Phuong national park of Ninh Binh, and mountain areas located to the north of the Chi Linh district of Hai Duong province. The highest suitability score areas were mostly located in the coastal areas to the east and south of Hanoi. These areas were chosen to have frequent vaccinations against the HPAI H5N1 disease in the Red River Delta. A total of 1137 communes, corresponding to 50.6% of total communes in the Delta, were selected for the alternative vaccination program, including provinces near the Gulf of Tonkin—Hai Phong, Thai Binh, Nam Dinh, Hai Duong, and the eastern parts of Hung Yen and Ha Nam provinces. These areas were also previously identified to have the highest probability of disease occurrence in the Delta [5]. Almost the entire areas of Hai Duong, Hai Phong, Thai Binh, and Nam Dinh provinces were identified as the focus areas for the alternative vaccination program except urban cores and mountains in the north of Hai Duong. Water bird production with free range farming was found to be the most intensive in the Red River Delta together with the Mekong Delta [55]. Thanh Oai, Thuong Tin, Ung Hoa, and Phu Xuyen districts of Hanoi were also identified as focus areas. These areas are famous for high-quality free-range duck meat provided to consumers in Hanoi.

The cost-effectiveness analysis indicated that the alternative vaccination program would better prevent the occurrence of the disease at lower costs associated with disease prevention than the national poultry vaccination program. Although the cost of the alternative vaccination program was estimated to be higher than that of the national poultry vaccination program (U.S. $5.92 million *vs*. U.S. $4.50 million), it resulted in reducing the rate of disease occurrence by 93.26% as compared to 61.66% of the national poultry vaccination program. As a result, this reduced the cost of immediate responses to the disease occurrence, including stamping out and emergency vaccination, government compensation, and farmers’ losses. This contributed to lower total costs associated with the alternative vaccination program as compared to the national poultry vaccination program (U.S. $6.92 million *vs.* U.S. $9.70). The results of the analysis suggested that Vietnam may face lower costs with the alternative vaccination program. 

Poultry production is much more complicated in reality. Producers may have different production cycles. Therefore, for the implementation of the alternative vaccination program, it is recommended that vaccination occurs at the commune level where the commune veterinary officers are required to monitor and vaccinate all poultry at recommended ages for every production cycle.

## 4. Conclusions

The national poultry vaccination program in the period 2006–2010, which implemented an annual two round vaccination plan for the entire geographical area of the Red River Delta, did not successfully control the disease. This study explored implications of an alternative vaccination program. This alternative vaccination program involves vaccination for every production cycle at the recommended poultry age in high risk areas. This alternative plan would have to be enacted at the local level for all production cycles.

This study identified the focus areas for the alternative vaccination program which were located mostly in the coastal areas to the east and south of Hanoi. A total of 1137 communes, corresponding to 50.6% of total communes in the Delta, were selected for the alternative vaccination program. The alternative vaccination program would have been less costly as compared to the national poultry vaccination program. Effectiveness analysis found that the alternative vaccination program would have been more successful in reducing the rate of disease occurrence from 61.66% rate of reduction (the national poultry vaccination program) to 93.26% rate of reduction (the alternative vaccination program). The cost analysis indicated that the alternative vaccination program would have saved the government and farmer resources because of lower total costs associated with prevention. Total losses imposed on both the government and farmers were higher for the national poultry vaccination program (U.S. $9.70 million) than for the alternative vaccination program (U.S. $6.92 million).
